# Level of immediate postpartum family planning utilization and the associated factors among postpartum mothers, Bole Sub-city, Addis Ababa, Ethiopia: institution based cross-sectional study

**DOI:** 10.1186/s12905-024-03038-7

**Published:** 2024-04-13

**Authors:** Abera Gezume, Ermias Wabeto, Helen Alemayehu

**Affiliations:** 1 Department of Public Health, Jinka University, Jinka, Ethiopia; 2 Summit Health Center, Woreda 05, Lemi-Kura sub-city, Addis Ababa Ethiopia

**Keywords:** Bole sub-city, Immediate, Postpartum, Family planning, Addis Ababa

## Abstract

**Background:**

The occurrence of pregnancy in the postpartum period poses a risk to women and their infants, and it also has increased risks of adverse health outcomes if a pregnancy happens less than two years after the preceding birth. Utilization of immediate postpartum family planning is a possible and simple way to reduce these unfavourable outcomes. However, only a small proportion of mothers use the service; but the reasons appear unclear. Thus, this study aimed to determine the level and factors associated with the utilization of immediate postpartum family planning in Bole sub-city, Addis Ababa, Ethiopia.

**Methods:**

A facility-based cross-sectional study was carried out from August 15 to September 15, 2022, among mothers who gave birth one year before the data collection period. A total of 425 mothers were selected with a systematic random sampling technique. A pretested and structured questionnaire was administered to collect data. Data entry and analysis were done by Statistical Package for Social Sciences 25. Chi-square, multicollinearity and Hosmer-Lemshaw model fitness tests were tested. The level of utilization was determined by descriptive statistics and the associated factors were determined by a binary logistic regression model, and presented with the adjusted odds ratios (AOR) with their respective 95% confidence intervals (95%CI). All statistical tests were conducted at a 5% level of significance.

**Results:**

Utilization of family planning method immediately after birth was 12.9% (95% CI = 11.3-14.5%), and it was statistically significantly associated with ages between 25 and 34 years (AOR = 5; 95% CI [1.38–18.41]) and 35 years and above (AOR = 6[1.47–25.70]), unfavourable attitude (AOR = 0.2[0.11–0.31]) and no counselling about immediate postpartum family planning during antenatal care visit (AOR = 0.43[0.20–0.89]).

**Conclusion and recommendations:**

The level of utilization of immediate postpartum family planning is low in the study area. To improve it, dealing with younger women, working to achieve a positive attitude amongst women towards immediate postpartum family planning, and incorporating counselling about postpartum family planning methods during antenatal care visits are all recommended.

**Supplementary Information:**

The online version contains supplementary material available at 10.1186/s12905-024-03038-7.

## Background

A woman is recommended to wait at least two years between the birth of the previous and conception of the succeeding baby to devote sufficient time to the previous child and to reduce the risk of maternal and child mortality by 30% and 10%, respectively [1–3]. When the next pregnancy occurs within one year after the previous childbirth, the risk of low birth weight, preterm delivery, and intrauterine growth retardation increases [4, 5]. Children born within 24 months after a previous birth are 60% more likely to die in the first year of life than those born later than two years [6]. Using postpartum family planning services is a possible way to reduce maternal and child morbidity and mortality [7, 8]. Globally, 80 million unintended pregnancies occurred in 2015 due to underutilization in the postpartum period [9]. Even though most of the postpartum women had the intention of using any kind of contraceptive methods within one year after childbirth, 70% could not access and/or employ them [1, 10].

Despite contraceptive utilization in Ethiopia is increasing from 6% in 2000 to 41% in 2019 [11, 12], almost half (47%) of the women got pregnant in less than two years between the previous childbirth and a succeeding pregnancy [13]. Therefore, creating an opportunity for a woman to start using contraceptive methods immediately after childbirth (within 48 h of delivery) was one of the strategies believed to increase the proportion of women using contraceptive methods to space the pregnancies and reduce unfavourable outcomes related to short spaced pregnancies and childbirths [14]; immediate postpartum family planning (IPPFP) refers to the counselling and provision of family planning methods to a woman within two days of giving birth to the last baby [15, 16].

If women start using the FP method during their immediate postpartum period (within 48 h of delivery), it reduces unmet needs which would bring much more benefits than starting at a later period. Integrating contraceptive service provision with early PNC is also worthwhile in saving time and costs for both the clients and service providers. To ensure the benefit and improve the access and utilization of IPPFP, the Ethiopian government and its development partners have made gains in policy development, training health workers and ensuring the availability of supplies [17, 18].

Regardless of the efforts made, the proportion of IPPFP utilization has been low in Ethiopia. According to studies conducted in some parts of the country, the utilization ranges from 13 to 39.2% and is positively associated with urban residence, attending secondary and tertiary school, having four or more antenatal care (ANC) visits in previous pregnancy, giving previous childbirth at a health facility, maternal knowledge of FP methods, partner involvement in decision making to use FP, maternal age (< 24 years), maternal age of 30–34 years, having ANC and Postnatal care (PNC) visits, previous history of FP utilization, and having an intention to limit childbirth and positive attitude towards family planning [19–24].

Evidence on the utilization of IPPFP is not exhausted or not well studied in the country. Besides this, the existing information is not consistent; showing varying proportions (13%) [24] to 39.2% [25] and inconsistent associated factors to guide interventions in Ethiopia. Moreover, substantial evidence concerning family planning utilization among early postpartum women is also lacking in Addis Ababa. Therefore, this study aimed to determine the level of immediate postpartum family planning (IPPFP) utilization and the associated factors in Bole Sub-city, Addis Ababa, Ethiopia.

## Methods

### The study area

The study was carried out in the Bole Sub-city, Addis Ababa, Ethiopia. The sub-city has an estimated total population of 221,006 with a one-to-one male-to-female ratio, and the estimated number of childbearing-age women is 51,494. Residents of the sub-city are served by 10 public health centres, 9 private hospitals and 91 private clinics in the sub-city.

### Study design and period

A facility-based cross-sectional study was conducted from August 15 to September 15, 2022.

### Source and study population

The source population was all postpartum women living in the sub-city and who gave birth one year before the data collection period, and the study population was all women who gave birth one year before data collection and who were selected to participate in the study.

### Sample size determination

A single population proportion formula was used to calculate sample size with the following assumptions: an expected 21.3% utilization of IPPFP [26], a 5% significance level and a 5% error tolerance. With this assumption, a sample size of 256 was computed. The sample size calculated for factors with a power of 80% was found to be lower than 256. After adding 10% to compensate for expected non-response and considering 1.5 design effects, the final sample size became 425 and this was used to determine both of the objectives.

### Sampling Procedure

Out of 10 public health centres in the Bole sub-city, four (Summit, Amoraw, Goro and Meri) were selected by applying a simple random sampling technique. The sample required from each health centre was proportionally allocated based on the health centre catchment and target population size. Then, women who gave birth one year before the data collection period and attended health centres for child and maternal health services were interviewed for socio-demographic characteristics, household economic status, access to health-related information and services and contraceptive utilization. The study participants were selected using a systematic random sampling technique with predetermined interval based on estimated client flows, a sample size required from each health facility and total data collection days.

### Data collection

Two midwives fluent in the Amharic language and one senior midwife supervisor from each health centre were trained in study overview, ways of approaching the women, communication, respecting the cultural norms of the women, the process of obtaining informed consent, administration of the questionnaire and keeping confidentiality. The questionnaire was adapted from the previous study [12] and contained sections to assess socio-demographic characteristics, economic status, access to health-related information and maternal services utilization, women’s decision-making power, partner involvement in decision-making and contraceptive utilization among postpartum women. Pretesting of the questionnaire was carried out at Gerji Health Center among twenty-one women who gave birth to a child within one year before the actual data collection. A modification was made to the questionnaire after pretesting and included the time of discharge from the health facility after child birth as a reference for those women who could not remember the exact time of starting contraceptive utilization. A discussion was held with the data collectors and supervisors for further clarity. The data were collected with a verbally-administered questionnaire which was delivered in person to all participants.

#### Variables

##### The dependent variable

The level of immediate postpartum family planning utilization.

##### Independent variables

Demographic characteristics (age, educational status, marital status, occupation, economy, number of children born, family support, decision-making power, number of living children and customs and beliefs), service factors (antenatal care, postnatal care, place of delivery, counselling on IPPFP, history of FP utilization, FP information, knowledge of FP, plan to space/limit birth and attitude towards FP).

#### Operational definitions

##### IPPFP utilization

It refers to the act of using a contraceptive method by a postpartum woman immediately after birth (within 48 h of delivery) [27].

##### Contraceptives

It is a method or drug of different kinds which can prevent unwanted or unplanned pregnancy [28, 29].

##### Modern contraceptive

Male and female sterilization, injectable, intrauterine devices (IUDs), contraceptive pills, implants, female and male condoms, the Standard Days Method, and emergency contraception were considered modern contraceptives [12].

##### Positive attitude

A woman who scored above the mean in attitude measuring questions was considered to have a positive attitude towards PPF [27].

##### Knowledgeable of FP

A mother was considered knowledgeable of FP if she correctly answered at least half of twelve knowledge-assessing questions [30].

##### Family planning

This is the ability of individuals and couples to anticipate and attain their desired number of children and the spacing and timing of their births through the use of contraceptive methods [31].

##### Wealth index

Relative measure of the household economy measured by household assets, and divided into five equal parts (quintiles); the poorest, poor, middle, rich and the richest.

##### Awareness of contraceptives

The respondents know any type of contraceptive method to space or limit pregnancy.

### Quality assurance

Before the actual data collection, 5% of the total sample was pretested at Gerji Health Center, and necessary corrections were made accordingly. Translation of the questionnaire from English to Amharic and back to English was undertaken by different individuals to check consistency. Supervision of data collection was conducted by the supervisor and the investigator. At the end of each data collection day, data were checked for completeness and consistency, and corrective measures were taken. An individual identification number was assigned to each questionnaire, and entry was made in SPSS-25 for Windows. After data entry, 10% of the sample was checked for correct entry and data cleaning was continued to the end of descriptive analysis.

### Data analysis

The data were analyzed with SPSS-25 for Windows. The economic status of the study participants was assessed by wealth index using household assets, housing conditions and household utilities via a principal component analysis (PCA). An asset owned by more than 97.5% or less than 2.5% of households was excluded during the data reduction process, and categorical variables with multiple options were transformed into binary variables [32]. In this regard, fourteen variables (type of latrine a household was using, an energy source for cooking, owning a computer, refrigerator, table with chairs, cooking materials, kerosene lamp, watch, bicycle, car, cash receipt from the safety net and other support, household enrollment in community-based health insurance, owning or living in charge-free home and flooring material of the dwelling) were used to generate the principal components [12]. Kaiser-Meyer-Olkin’s measure of sampling adequacy and Bartlett’s test of sphericity were 0.91 and less than 0.001 (significant), respectively. The total variance explained by components with eigenvalues greater than one was 63.9%. Then, economic status was grouped into quintiles; the poorest, poor, middle, rich and the richest. The knowledge of mothers was tested by twelve binary questions and a mother who answered half and above was considered knowledgeable [27, 30]. Knowledge of FP, place of delivery, birth interval, information on FP, customs and occupation violated the chi-square assumption (had an expected cell count less than 5%), and PNC visits and the number of living children were incomplete and, therefore, excluded from a logistic regression model. Attitude towards family planning was tested by seven questions asking the level of agreement about FP using a 5-point Likert scale of 1 = strongly disagree to 5 = strongly agree. The total score ranged from 7 to 35 and the mean was used as a cut-off. The mean score was 28 and a mother who scored above the mean score was taken as having a positive attitude, the remainder characterized as having a negative attitude [27, 30]. The reliability of the questionnaire was tested with Cronbach’s alpha and found to be 0.72.

Descriptive statistics was used to compute the level of IPPFP and logistic regression was undertaken to identify the factors statistically significantly associated with IPPFP utilization among postpartum mothers. A multi-collinearity test was carried out to check the interrelationship between independent variables at a variable inflation factor less than ten or tolerance greater than 0.1. No variables were interdependent and the highest variable inflation factor value was 1.9. Findings of descriptive analysis were presented with frequency and percentage, mean or median and the regression outputs were presented with COR and AOR with their respective 95% CI. A statistical significance level was set when a p-value was less than 0.05 in multivariable analyses.

## Results

### Socio-demographic characteristics of the respondents

Four hundred and twenty-five mothers who gave birth within one year prior to the data collection period were approached and successfully responded to the interview; the response rate was 100%. The mean age of the respondents was 28 (± 6) years with minimum and maximum ages of 18 and 48 years, respectively. More than three-fourths (80.5%) of the study participants reported that they can write and read and one-fifths (21.2%) completed secondary school. About two in ten (18.4%) of the respondents were from the poorest households (Table 1).

**Table 1:** Socio-demographic characteristics of the respondents, Bole sub-city, Addis Ababa, Ethiopia, 2022

Characteristics	Frequency (*n* = 425)	Percentage
Age		
Less than or equal to 24 years	129	30.4
25–34 years	217	51.1
Greater or equal to 35 years	79	18.6
Educational status		
Illiterate	83	19.5
Primary	72	16.9
Secondary	90	21.2
Diploma	78	18.4
Degree	88	20.7
Master’s and above	14	3.3
Marital status		
Married	344	80.9
Single	62	14.6
Others	19	4.5
Occupation		
Self employed	107	25.2
Government employed	125	29.4
Housewife	104	24.4
Daily laborer	64	15.1
Non-governmental organization	12	2.8
Other	13	3.1
Family size		
Less than 5	316	74.4
Five and above	109	25.6
Partners education		
Illiterate	61	14.3
Primary	36	8.4
Secondary	126	29.6
Diploma	68	16.0
Degree	118	27.7
Master’s and above	17	4.0
Occupation of partner		
Self employed	200	47.0
Government employed	122	28.7
Daily laborer	73	17.2
Non-government	20	4.7
Other	10	2.4
Children ever born		
Three and below	410	96.4
Four and above	15	3.6
Household wealth status		
The poorest	78	18.4
Poor	96	22.6
Middle	91	21.4
Rich	83	19.5
The richest	77	18.1

### Information on contraceptive methods, maternal awareness and attitude towards FP

The respondents had access to information regarding contraceptive methods from different sources and heard about family planning (contraceptive) methods from various information sources. Most (94.8%) heard from health workers while 81.6%, 68.2%, 54.8% and 49.9% got information from the mass media, friends, family members and schools, respectively. Regarding awareness of the types of contraceptive methods, 98.6% of study participants knew about pills. Three-fourths (76.7%) mentioned that they knew emergency pills could be used as a family planning method. Condoms as a family planning method were reported by 96% of respondents and an intra-uterine device was reported by 87.8% of respondents. Implants and injectables were mentioned as family planning methods by 96.5% and 97.9% of study participants, respectively. Calendar, lactation amenorrhea, permanent and withdrawal methods were reported by 33.6%, 30.4%, 18.6% and 10.6% of respondents, respectively. All of the respondents perceived that postpartum family planning can prevent unwanted pregnancy, maternal illness and death, and limit and space births. Almost all of the study participants mentioned that fertility can immediately resume after cessation of contraceptives of a given type. The majority of them (85.9%) believed that a woman can start using contraceptives before menstruation resumes after giving birth to a baby, and (99.3%) reported that a woman can access family planning methods from public health facilities. More than half of the respondents (53.9%) had a positive attitude towards IPPFP specifically and FP in general.

### Reproductive history and maternal health service utilization

Most of the study participants (94.8%) received ANC care during their most recent child pregnancy and more than one-third (40%) of them had four or more ANC visits while the remaining 39.3% and 14.6% had three and two visits, respectively. About 95% of respondents reported that they gave birth to their recent child at health facilities; 52.9% at public hospitals, 38.1% at public health centres and 4.2% at private hospitals.

All of the respondents visited health facilities at least once after the birth of the last child for child immunization (65.9%), PNC (31.5%) or other purposes (2.6%). The median number of children ever born to a woman was 2 (IQR = 1); 51.8% of respondents gave birth to more than one child, and the minimum and maximum number of children born to a woman was one and six, respectively. The median age of the last child was 7 (IQR = 3) months, and 41.7% of them were below six months old. Three-fourths of the respondents reported that their menses resumed; 41.4% and 31.0% reported that they saw menstruation in less than three and four to six months after the delivery of their recent child, respectively. More than three-quarters of respondents disclosed that they started sexual intercourse. About three-fourths (74%) of them started later than six weeks after delivery of the baby.

### Contraceptive-related service utilization

All of the respondents had heard about family planning methods during child health services (60.7%), health-related programs (34.6%) and/or health facility visits (4.7%). Half (50.1%) and about six in ten (58.8%) of the respondents were counselled about family planning during their recent ANC visits and immediately after giving birth to the last child. More than two-thirds (70.1%) used family planning methods of various types. About 13% (12.9%; 95%CI= [9.7-16.1%]) of respondents started using contraceptives immediately or within 48 h of childbirth while half (50.8%) started between three and 90 days. More than half (59.1%) of respondents accessed/procured contraceptive methods from public health facilities.

The respondents who did not start using family planning methods disclosed their reasons; it is not socially acceptable to take contraceptives immediately after childbirth (30.8%), not living together with a husband or partner (14.4%), pregnancy can’t happen immediately after childbirth (10.6%), perceived effect on breast milk (8.9%), fear of side effects during the postpartum period (5.4%) and others (2.4%).

### Factors associated with the utilization of IPPFP

Ten variables (maternal age, maternal education, partner education, family size, children ever born to a woman, attitude towards IPPFP, household living standard, counselling about FP during ANC visit, counselling about FP during or immediately after birth and maternal decision power) were used in the bi-variable logistic regression model. All variables had a p-value less than 0.2 in the bi-variable analysis and were considered in the multivariable logistic regression model. Three variables; age of the respondents, maternal attitude towards IPPFP and counselling about FP during the recent ANC visits were statistically significantly associated with the utilization of IPPFP. As compared to women aged 24 years and below, those aged 25 to 34 years and 35 and above had five (AOR = 5.0; 95%CI [1.4–18.4]) and six (AOR = 6.2; 95%CI [1.5–25.7]) times increased odds of using IPPFP, respectively. The women who had an unfavourable attitude towards IPPFP had 80% (AOR = 0.2; 95%CI [0.1–0.31]) reduced likelihood of using IPPFP than their counterparts. As compared to women who were counselled about family planning methods during ANC visits, their counterparts had 60% decreased odds of using IPPFP (AOR = 0.4; 95%CI [0.2–0.8]) (Table 2).

**Table 2:** Bi-variable and multi- variable analysis of factors associated with IPPFP utilization in Bole Sub-city, Addis Ababa, Ethiopia 2022

Variable category	IPPFP Utilization		COR (95%CI)	AOR (95%CI)
	No (*n* = 370)	Yes (*n* = 55)		
Age of respondents				
<= 24 years	126	3	1	1
25–34 years	178	39	9.20(2.78–30.44)	5.0(1.4–18.4)**
>= 35 years	66	13	8.27 (2.28–30.06)	6.2(1.4–25.7)**
Maternal Education				
Cannot write and read	60	1	0.30(0.10–0.82)	1.1(0.3–4.5)
Can write and read	310	54	1	1
Family size				
Less than five members	284	32	1	1
Five and above members	86	23	2.40(1.32–4.27)	1.4(0.7–2.8)
Children born to a woman				
Three and above	57	17	2.51(1.32–4.75)	2.1(0.7–5.8)
Below three	314	38	1	1
Partner Education				
Cannot write and read	60	1	0.09(0.01–0.70)	0.4(0.1–4.1)
Can write and read	310	54	1	1
Household economy				
The poorest	75	3	0.11(0.03–0.37)	0.9(0.2–4.2)
Poor	92	4	0.12(0.04–0.36)	0.6(0.1–2.2)
Middle	83	8	0.26(0.11–0.62)	0.5(0.2–1.6)
Rich	64	19	0.79(0.39–1.62)	0.7(0.3–1.7)
The richest	56	21	1	1
Frequency of ANC visit				
Below four visits	232	23	1	1
Four and above visits	138	32	2.34(1.32–4.16)	1.1(0.5–2.3)
Counselling for FP during ANC visit				
No	200	12	0.24(0.12–0.46)	0.4(0.2–0.8)**
Yes	170	43	1	1
Counselling for FP during/immediately after giving birth				
No	162	13	0.40(0.21–0.76)	0.5(0.3–1.2)
Yes	208	42	1	
Attitude towards IPPFP				
Unfavorable attitude	194	2	0.03 (0.01–0.14)	0.2(0.1–0.3)**
Positive	176	53	1	1
Women decision power on using IPPFP				
Cannot decide on using IPPFP	156	6	0.17(0.10–0.40)	0.7(0.2-2.0)
Can decide	214	49	1	1

** The variables statistically significantly associated with the dependent variable

## Discussion

The current study was carried out in Bole Sub-City, Addis Ababa, Ethiopia, to determine the level of immediate postpartum family planning utilization and the associated factors among mothers who gave birth within one year before the data collection period. The level of IPPFP utilization was 12.9% (95% CI = 11.3-14.5%) and statistically significantly associated with maternal age (as age increases, the likelihood of using IPPFP increases), good maternal attitude towards FP and counselling about postpartum family planning during ANC visits.

Our study found that 12.9% of postpartum women used contraceptives of any type immediately after giving birth to their last child. This is consistent with the findings revealed by studies conducted at Saint Paul Millennium Medical College, Addis Ababa, 2019; 12.7% [24] and in Somalia (12.7%) [33]. This indicates the utilization remained similar across time in the city and country, this calls for strengthening interventions to improve the utilization. But, the finding is lower than what was witnessed by the study carried out in the United States of America (USA); 49% [34]. The difference may be due to variation in attitude towards IPPFP; half of the respondents in the current study had unfavourable attitudes whereas most of the participants in the USA had positive attitudes. The finding implies that IPPFP utilization is an antecedent of good attitude; thus, efforts should be paid to improve maternal attitudes towards IPPFP in the study area.

The current finding is also lower than the findings shown by a study in Kenya; 50% [35]. The inconsistency may be due to the difference in the participants concerning their HIV infection status; HIV-positive women participated in Kenya. It is obvious that HIV-infected women were strongly advised to take contraceptives to limit the number of children unless they had special conditions. The level of IPPFP is found to be lower than the level in Jima (53%) [15], Sidama; 21.6% [30], North Shoa; 21.3% [27], Hawassa teaching and referral hospital; 25.4% [36], in four regions of Ethiopia (Tigrai, Amhara, South and Oromia); 19.7% ( [37] and in Ethiopia; 39% [25]. The possible reason for inconsistent findings may be that the presence of intervention programs (which intended to increase utilization of LARC immediately after childbirth among rural women in other study areas) might result in intense counselling and social mobilization activities and, in turn, increased uptake of contraceptives. Another possibility may be those study settings are relatively easy to cascade to health services with different modalities like home-to-home visits by the health development army, health extension workers and others; as a result, repeated counselling and service provision may be undertaken.

When the age of the woman increases, the likelihood of using IPPFP increases. As compared to women aged 24 years and below, those aged 25 to 34 years and 35 years and beyond had five and six times increased odds of using IPPFP, respectively. The findings agree with the study carried out in humanitarian settings in Pakistan in 2021 [33], but disagree with findings from Somalia and the Democratic Republic of Congo [33], Kenya [38], Gondar [39] and North Shoa [27]. The difference may be due to the older participants in the current study area having achieved the number of children and family size they planned to have, or the younger respondents in other studies facing previous unplanned pregnancies who therefore decided to use IPPFP. This shows that the younger women need attention and targeted counselling and education concerning IPPFP. Age appropriate and inclusive counselling and education about contraceptive plays crucial role in improving PPFP utilization.

Counselling about postpartum family planning during ANC visits enhanced the utilization of IPPFP in a current study area. As compared to women who were counseled for PPFP during recent ANC visits, their counterparts had 60% reduced odds of using IPPFP. This is consistent with findings shown by studies conducted in rural Kenya [38], Systematic Review and Meta-analysis in Ethiopia [40], Jimma [15], Sidama [30], Southern Ethiopia [41] and public health facilities of Ethiopia [25]. This shows that the ANC visit or schedule can be used as the key strategy to convey information regarding IPPFP to the target groups and counselling about postpartum family planning should be ensured during ANC visits. If the women get information about PPFP during ANC visits, they will have adequate time to think, seek advice from the partners and family members to choose contraceptive types and time when to start after childbirth.

Having a favourable (positive) attitude towards postpartum family planning was statistically significantly associated with IPPFP uptake in a study area; respondents with unfavourable attitudes had 80% reduced likelihood of using IPPFP than respondents on the other side. Consistent with studies carried out in North Shoa [27] and Saint Paul Millennium Medical College in Addis Ababa [24]. A good attitude motivates the utilization of IPPFP; therefore, behavioural change interventions like experience sharing are required to improve it among women to enhance their IPPFP utilization because good attitude is the precedent factor that motivates the behaviour to happen and sustain.

### Limitations and strengths

The time interval between giving birth to their last child and starting to use contraceptives was estimated by the respondents, and thus recall bias may be introduced. The wider CI tells a smaller sample size and less stable power; thus, precaution is required. Pretesting the questionnaire before the actual data collection, training the research assistants, conducting close supervision, covering a wide study area (the sub-city), and taking the necessary data quality assurance methods are the strengths of the current study.

## Conclusion and recommendations

The level of immediate postpartum family planning utilization is low in the study area, and it was associated with maternal age, counselling about postpartum family planning during ANC visits, and maternal attitude towards immediate postpartum family planning. To improve immediate postpartum family planning utilization among postpartum mothers in the study area, giving attention to younger women during counselling, health education and other health-related programs, integrating counselling about family planning methods with prenatal services, and improving the attitude of postpartum women towards IPPFP are all recommended.

### Electronic supplementary material

Below is the link to the electronic supplementary material.

Supplementary Material 1

Supplementary Material 2

## Data Availability

The datasets analyzed during the current study are available in the Medley repository; https://data.mendeley.com/datasets/xr4kvk9pb9/1.

## Abbreviations

ANC: Antenatal Care
AOR: Adjusted Odds Ratio
CI: Confidence Interval
COR: Crude Odds Ratio
IPPFP: Immediate Postpartum Family Planning
IQR: Inter Quartile Range
LARC: Long Acting Reversible Contraceptive
PCA: Principal Component Analysis
USA: United States of America
