# Supplementary material for: Level of immediate postpartum family planning utilization and the associated factors among postpartum mothers, Bole Sub-city, Addis Ababa, Ethiopia: institution based cross-sectional study
Source: BMC Womens Health. 2024 Apr 13;24:237. doi: 10.1186/s12905-024-03038-7 (PMC11015549; doi:10.1186/s12905-024-03038-7)
Supplement: Supplementary file 1 — Supplementary Material 1 [file 12905_2024_3038_MOESM1_ESM.docx]

# Questionnaire

**Section -1:** **Socio -demographic characteristics of the participants**

| Q.no | Questionnaires | Response | Skip |
| --- | --- | --- | --- |
| 1 | How old are you? (Your Age ) | ……… |  |
| 2 | Educational status? | 1. Cannot write and read (Illiterate) 2. Literate | If 1, go to Q4 |
| 3 | If literate for Q 2 above, what grade did you complete? | ___________________ |  |
| 4 | What is your marital status? | 1. Married  2. Single  3. Divorced  4. Widowed |  |
| 5 | What is your current occupation? | 1. Self-employee  2.Government employee  3. House wife  4. Daily laborer  5. NGO  6. Others |  |
| 6 | Educational status of your partner? | 1. Cannot write and read (Illiterate) 2. Literate | If 1, go to Q8 |
| 7 | If literate for Q 6 above, what grade did you complete? | ___________________ |  |
| 8 | What is your husband occupation? | 1. Private employee  2. Daily laborer  3 Government employee  4. Merchant  5 . NGO  6. Others……… |  |
| 9 | Total number of family members | ___________________________ |  |
| 10 | Where do you mainly get water for your household from? | 1. Tap inside my house 2. Tap in my own yard/plot 3. Tap from neighbor 4. Public stand 5. Dug well 6. Protected spring 7. Unprotected spring 8. River |  |
| 11 | What kind of toilet facility do members of your household usually use? | 1. Flush toilet ( flush to sewer, septic tank or pit) 2. Ventilated improved pit latrine 3. Simple pit latrine 4. Share with neighbor 5. No toilet facility |  |
| 12 | What type of fuel does your household mainly use for cooking? | 1. Electricity 2. LPG 3. Natural gas 4. Biogas 5. Kerosene 6. Charcoal 7. Wood 8. Straw/shrubs/grass 9. Agricultural crop 10. Animal dung 11. No food cooked in household |  |
| 13 | Does your household own the following materials | 1. Electric? 0. No 2. No 2. A radio? 0. No 2. No 3. Home Telephone? 0. No 2. No 4. A computer? 0. No 2. No 5. A refrigerator? 0. No 2. No 6. A table? 0. No 2. No 7. A bed with mattress 0. No 2. No 8. An electric Mitad? 0. No 2. No 9. A kerosene lamp? 0. No 2. No |  |
| 14 | Does any member of your household own | 1. Watch? 0. No 1. Yes 2. Mobile phone? 0. No 1. Yes 3. A bicycle 0. No 1. Yes 4. A motorcycle? 0. No 1. Yes 5. Animal drawn cart? 0. No 1. Yes 6. Car or truck? 0. No 1. Yes 7. A bajaj 0. No 1. Yes |  |
| 15 | Does any member of this household have a bank account or microfinance savings account? | 1. No 2. Yes |  |
| 16 | Is your household receiving cash or food from the Safety Net Program? | 1. No 2. Yes |  |
| 17 | Is your household enrolled in a Community Based Health Insurance scheme? | 1. No 2. Yes |  |
| 18 | Does your household own the dwelling or occupy dwelling free of charge? | 1. No 2. Yes |  |
| 19 | Floor of the dwelling your household | 1. Sand/earth/dung 2. Wooden 3. Carpets 4. Cemented 5. Ceramic |  |
| 20 | The material roof of your dwelling covered with | 1. No roof 2. Cardboard 3. Hatch/grass/leaf 4. Wooden 5. Iron/metal 6. Calamine/cement fiber 7. Cement 8. Roofing shingles |  |
| 21 | The material wall of your dwelling covered with | 1. No wall 2. Wooden only 3. Cardboard 4. Stone with mud 5. Plastics 6. Cemented stones 7. Cemented blocks 8. Bricks |  |

**Section 2: Source of information and knowledge of the post-partum women on contraceptive methods**

| Q.no | Questions | | Response | Skip |
| --- | --- | --- | --- | --- |
| 1 | Have you heard of any contraceptive (FP) methods? | | 1. Yes 2. No | If 2, go to Q4 |
| 2 | What is your source of information about FP?  ( Multiple response is possible ) | | 1. Health facility  2. Mass media (TV, Radio, etc…)  3. Health extension workers/workers  4. friends  5. family  6. churches  7. schools  8. Others (specify)………….. |  |
| 3 | Which type of Family Planning do you know?  (Multiple response is possible) | | 1. Oral pill  2. Emergency pill  3. Condoms  4. IUD  5. Implant  6. Injection  7.Sterilization (female and male)  8. LAM  9. Calendar method  10. Standard day method  11. With drawl method  12. Others |  |
| 4 | From where someone (women) can obtain the FP method?  (Multiple response is recommended ) | | 1. Public health facility  2. Private health facility/clinic  3. Pharmacy/Drug shop  4. Others (specify) ………… |  |
| Questionnaires measuring Knowledge of women towards PPFP | | | |  |
| 5 | | Do you believe that PPFP helps to prevent unwanted pregnancies | 1. Yes 0. No |  |
| 6 | | Do you believe that PPFP helps to prevent possible maternal death and illness | 1. Yes 0. No |  |
| 7 | | Do you believe that PPFP helps to limiting number of children | 1. Yes 0. No |  |
| 8 | | Do you believe that PPFP helps to space between child’s | 1. Yes 0. No |  |
| 9 | | Do you believe that Fertility resumed after stopping contraceptive | 1. Yes 0. No |  |
| 10 | | Do you believe that Exclusive breast-feeding used as FP | 1. Yes 0. No |  |
| 11 | | Do you believe that woman can start contraception before the menstruation begins after delivery | 1. Yes 0. No |  |

**Section -3: Attitude of post-partum women towards Contraceptive**

| Q.no | Questionnaires | Response | Skip |
| --- | --- | --- | --- |
| 12 | Husband must have to decide whether the wife has to use family planning or not | 1. Strongly disagree  2. Disagree  3. Neutral  4. Agree  5. Strongly agree |  |
| 13 | Discussing PPFP use with partner is good | 1. Strongly disagree  2. Disagree  3. Neutral  4. Agree  5. Strongly agree |  |
| 14 | Using contraceptive can cause infertility | 1. Strongly disagree  2. Disagree  3. Neutral  4. Agree  5. Strongly agree |  |
| 15 | Men should share the responsibility of family planning use. | 1. Strongly disagree  2. Disagree  3. Neutral  4. Agree  5. Strongly agree |  |
| 16 | Post-partum contraceptive helps a mother to regain her strength before her next baby | 1. Strongly disagree  2. Disagree  3. Neutral  4. Agree  5. Strongly agree |  |
| 17 | Encourage your friends to use PPFP | 1. Strongly disagree  2. Disagree  3. Neutral  4. Agree  5. Strongly agree |  |
| 18 | Un married/ women not living together with partner can use contraceptive | 1. Strongly disagree  2. Disagree  3. Neutral  4. Agree  5. Strongly agree |  |
| 19 | Using contraceptive deviates from cultural beliefs | 1. Strongly dis agree  2. Disagree  3. Neutral  4. Agree  5. Strongly agree |  |
| 20 | Your Religion forbids contraceptive use | 1. Strongly dis agree  2. Disagree  3. Neutral  4. Agree  5. Strongly agree |  |
| 21 | Discussing about contraceptive with family members is important | 1. Strongly dis agree  2. Disagree  3. Neutral  4. Agree  5. Strongly agree |  |

**Section -4: Family Planning and other health services utilization, and sexual and reproductive health characteristics of post-partum women utilization in the postpartum period.**

| Q.no | Questionnaires | Response | Skip |
| --- | --- | --- | --- |
| 22 | Did you use a FP method within 12 months after delivery of you’re the most recent child? | 1. Yes  2. No | If No (2), go to Q25 below |
| 23 | If yes to 21, what method did you use?  (pleas Mention All that you used ) | . Oral pill  2. Emergency pill  3. Condoms  4. IUD  5. Implant  6. Injection  7.Sterilization (female and male)  8. LAM  9. Calendar method  10. Standard day method  11. With drawl method  12. Others |  |
| 24 | From where did you get the FP method? | 1. Government health facility 2. Private health facility 3. NGO donation 4. Pharmacies/drug venders |  |
| 25 | When did you start using the FP method after delivery of the recent child? | 1. -------------days or ____ weeks 2. Before going to home from health facility where gave birth |  |
| 26 | If you did not use FP method, in the first 12 months why did not use?  (Multiple response is possible) | 1. Fear of side effects 2. Want to deliver soon 3. Fear of change in breast milk by FP methods 4. No knowledge about FP  5. Spousal not present near/No sexual contact  6. Feeling of not susceptible to Pregnancy due to breastfeeding 7. Feeling of not at risk of pregnancy due to amenorrhea 8. Absence of FP methods in my residence 9. Absence of chosen method  10. Not acceptable by culture  11. Not acceptable by religion  12. Others, specify------------- |  |
| 27 | Currently do you use FP method | 1. Yes 2. No | If no (2) got to Q 29 below |
| 28 | If yes for Q. 27, which method of family planning you use? | 1. Pill 2. IUD 3. Inject able 4. Implant  5. Condom  6. Others (specify) ……………… |  |
| 29 | If not for Q 27, why? | Fear of side effects 2. Want to deliver soon 3. Fear of change in breast milk by FP methods 4. No knowledge about FP  5. Spousal not present near/No sexual contact  6. Feeling of not susceptible to Pregnancy due to breastfeeding 7. Feeling of not at risk of pregnancy due to amenorrhea 8. Absence of FP methods in my residence 9. Absence of chosen method  10. Not acceptable by culture  11. Not acceptable by religion  12. Others, specify------------- |  |
| 30 | Did you attend ANC clinic for your recent child | 1. Yes 2. No | If no (2), go to Q 32 below |
| 31 | If yes for Q. 30 How many times did you attend the ANC clinic | 1. One visit 2. Two visit 3. Three visit 4. Four and above visit |  |
| 32 | Did you counseled for FP during ANC visit | 1. Yes 2. No |  |
| 33 | Where did you delivered your recent child? | 1. Public hospital 2. Private hospital 3. Health center 4. Private clinics 5. Home | If home (5), go to Q 35 below |
| 34 | Have you been counseled for FP during/immediately delivery? | 1. Yes  2. No |  |
| 35 | Did you visit health facility after giving recent birth? | 1. Yes 2. No | If no (2), go to Q 37 below |
| 36 | Reason for visiting health facility after giving birth | 1. For child immunization 2. For post natal care 3. To get treatment 4. For family planning 5. Others specify………. |  |
| 37 | When have you been counseled for Family planning | 1. Before first delivery 2. After first delivery 3. During the second pregnancy 4. When I come For other health services |  |
| 38 | Would you tell me your total pregnancy you experienced? | _______________ |  |
| 39 | Would you tell me your total number of children you gave birth to? | _______________ |  |
| 40 | How old is your youngest child? | ________days  Or ______ weeks or ____months |  |
| 41 | How long did it take to give the birth to the youngest child after the birth of previous birth? | 1. First birth 2. < 2 years   3. 2-3 years  4. > 3 years |  |
| 42 | Your menses resumed after recent child | 1. Yes 2. No | If no (2), go to Q 44 below |
| 43 | How long in months did it take to your menses resume after birth of your recent child? | 1. < 3 months 2. 4-6 months 3. 7-9 months 4. Others (specify)... |  |
| 44 | Have you started sexual intercourse after you gave birth to the recent birth? | 1. Yes 2. No | If no (2), go to Q 46 below |
| 45 | How long did you stay to start sexual intercourse after the recent child birth? | 1. < 6 weeks 2. 6 weeks-3 months 3. 4-6 months 4. 7-9 months |  |
| 46 | Have you currently started using contraceptive? | 1. Yes 2. No |  |
| 47 | If yes for Q.44, When did you start using contraceptive? | 1. Immediate after birth (within 48 hours) 2. 6 weeks-3 months 3. 4-6 months 4. 7 month and above |  |
