# Supplementary material for: Level of immediate postpartum family planning utilization and the associated factors among postpartum mothers, Bole Sub-city, Addis Ababa, Ethiopia: institution based cross-sectional study
Source: BMC Womens Health. 2024 Apr 13;24:237. doi: 10.1186/s12905-024-03038-7 (PMC11015549; doi:10.1186/s12905-024-03038-7)
Supplement: Supplementary file 2 — Supplementary Material 2 [file 12905_2024_3038_MOESM2_ESM.docx]

STROBE Statement—checklist of items that should be included in reports of observational studies

|  | Item No. | Recommendation | Page  No. | Relevant text from manuscript | |
| --- | --- | --- | --- | --- | --- |
| **Title and abstract** | 1 | (*a*) Indicate the study’s design with a commonly used term in the title or the abstract | 2 | A facility-based cross-sectional study was carried out from August 15 to September 15. | |
|  |  | (*b*) Provide in the abstract an informative and balanced summary of what was done and what was found | 2-3 | The level of immediate post-partum family planning utilization was 12.912.9% (95% CI= 11.3%-14.5%) |  |
| Introduction | | |  |  |  |
| Background/rationale | 2 | Explain the scientific background and rationale for the investigation being reported | 4-5 | However, substantial evidence on family planning utilization among early postpartum women is lacking | |
| Objectives | 3 | State specific objectives, including any prespecified hypotheses | 5 | Therefore, this study aimed to determine the level of immediate postpartum family planning. | |
| Methods | | |  |  |  |
| Study design | 4 | Present key elements of study design early in the paper | 6 | A facility-based cross-sectional study was conducted. | |
| Setting | 5 | Describe the setting, locations, and relevant dates, including periods of recruitment, exposure, follow-up, and data collection | 6 | The study was carried out in the Bole sub-city. | |
| Participants | 6 | (*a*) *Cohort study*—Give the eligibility criteria, and the sources and methods of selection of participants. Describe methods of follow-up  *Case-control study*—Give the eligibility criteria, and the sources and methods of case ascertainment and control selection. Give the rationale for the choice of cases and controls  *Cross-sectional study*—Give the eligibility criteria, and the sources and methods of selection of participants | 6-7 | **It was a cross-sectional study**; The source population was all postpartum women living in the sub-city who gave birth one year before the data collection period.  The source population and eligibility criteria stated under study population section. The selection was stated under sampling procedure | |
|  |  | (*b*) *Cohort study*—For matched studies, give matching criteria and number of exposed and unexposed  *Case-control study*—For matched studies, give matching criteria and the number of controls per case |  | NA |  |
| Variables | 7 | Clearly define all outcomes, exposures, predictors, potential confounders, and effect modifiers. Give diagnostic criteria, if applicable | 8 | The dependent variable was the level of immediate postpartum family planning utilization. | |
| Data sources/ measurement | 8* | For each variable of interest, give sources of data and details of methods of assessment (measurement). Describe comparability of assessment methods if there is more than one group | *8* | The data were collected via the interviewer-administered questionnaire. | |
| Bias | 9 | Describe any efforts to address potential sources of bias | 8 | The pretesting of the questionnaire was conducted among twenty women. | |
| Study size | 10 | Explain how the study size was arrived at | 6 | A **single population proportion formula was used to calculate the**sample size. | |

Continued on next page

| Quantitative variables | 11 | Explain how quantitative variables were handled in the analyses. If applicable, describe which groupings were chosen and why |  | 10 | The mean age of the respondents was 28 (±6) years. | | |
| --- | --- | --- | --- | --- | --- | --- | --- |
| Statistical methods | 12 | (*a*) Describe all statistical methods, including those used to control for confounding |  | 10 | Multi-variable logistic regression was undertaken to identify the factors associated with IPPFP utilization. | | |
|  |  | (*b*) Describe any methods used to examine subgroups and interactions |  | 10 | A multi-collinearity test was carried out to check the interrelationship between independent variables. | | |
|  |  | (*c*) Explain how missing data were addressed |  |  |  |  | |
|  |  | (*d*) *Cohort study*—If applicable, explain how loss to follow-up was addressed  *Case-control study*—If applicable, explain how matching of cases and controls was addressed  *Cross-sectional study*—If applicable, describe analytical methods taking account of sampling strategy |  | 10 | Kaiser-Meyer-Olkin measure of sampling adequacy and Bartlett's test of Sphericity were 0.91 and less than 0.001 (significant), respectively. The total variance explained by components with eigenvalues greater than one was 63.9%. | | |
|  |  | (*e*) Describe any sensitivity analyses |  | 10 | The model fitness was checked by Hosmer-Lemshaw test at p-value greater than or equals to 0.05. | | |
| Results | | |  | |  | |  |
| Participants | 13* | (a) Report numbers of individuals at each stage of study—eg numbers potentially eligible, examined for eligibility, confirmed eligible, included in the study, completing follow-up, and analysed |  | 11 | Four hundred and twenty-five mothers who gave birth within one year before the data collection period successfully responded to the interview. | | |
|  |  | (b) Give reasons for non-participation at each stage |  |  | N/A |  | |
|  |  | (c) Consider use of a flow diagram |  |  | Cross-sectional study | | |
| Descriptive data | 14* | (a) Give characteristics of study participants (eg demographic, clinical, social) and information on exposures and potential confounders |  | 11 | The mean age of the respondents was 28 (±6) years (Table 1). | | |
|  |  | (b) Indicate number of participants with missing data for each variable of interest |  |  | No missing value |  | |
|  |  | (c) *Cohort study*—Summarise follow-up time (eg, average and total amount) |  |  | NA |  | |
| Outcome data | 15* | *Cohort study*—Report numbers of outcome events or summary measures over time |  |  | *NA* |  | |
|  |  | *Case-control study—*Report numbers in each exposure category, or summary measures of exposure |  |  | *NA* |  | |
|  |  | *Cross-sectional study—*Report numbers of outcome events or summary measures |  | 13 | About 13% (12.9%; 95%CI= [9.7%-16.1%]) of the respondents started using immediately or within 48 hours of childbirth | | |
| Main results | 16 | (*a*) Give unadjusted estimates and, if applicable, confounder-adjusted estimates and their precision (eg, 95% confidence interval). Make clear which confounders were adjusted for and why they were  included | 13 |  | About 13% (12.9%; 95%CI= [9.7%-16.1%]) of the respondents started using immediately or within 48 hours of childbirth | | |
|  |  | (*b*) Report category boundaries when continuous variables were categorized |  |  | NA |  | |
|  |  | (*c*) If relevant, consider translating estimates of relative risk into absolute risk for a meaningful time period |  |  | NA |  | |

Continued on next page

| Other analyses | 17 | Report other analyses done—eg analyses of subgroups and interactions, and sensitivity analyses |  |  | *NA* |
| --- | --- | --- | --- | --- | --- |
| Discussion | | |  | |  |
| Key results | 18 | Summarise key results with reference to study objectives |  | 14 | The level of IPPFP utilization was 12.9% (95% CI= 9.5%-16.1%) |
| Limitations | 19 | Discuss limitations of the study, taking into account sources of potential bias or imprecision. Discuss both direction and magnitude of any potential bias | 17 |  | The women were asked to remember the length of ours after delivery to start PPFP, and recall bias may be introduced |
| Interpretation | 20 | Give a cautious overall interpretation of results considering objectives, limitations, multiplicity of analyses, results from similar studies, and other relevant evidence |  | 17 | The level of immediate postpartum family planning utilization among postpartum mothers is low in the study area. |
| Generalisability | 21 | Discuss the generalisability (external validity) of the study results |  | 17 | The level of immediate postpartum family planning utilization among postpartum mothers is low in the study area and in Ethiopia as well. |
| Other information | |  |  | |  |
| Funding | 22 | Give the source of funding and the role of the funders for the present study and, if applicable, for the original study on which the present article is based |  | 17 | No fund was obtained |

*Give information separately for cases and controls in case-control studies and, if applicable, for exposed and unexposed groups in cohort and cross-sectional studies.

**Note:** An Explanation and Elaboration article discusses each checklist item and gives methodological background and published examples of transparent reporting.
